# FDG PET texture indices as imaging biomarkers for epidermal growth factor receptor mutation status in lung adenocarcinoma

**DOI:** 10.1038/s41598-023-34061-7

**Published:** 2023-04-25

**Authors:** Mariko Ishimura, Takashi Norikane, Katsuya Mitamura, Yuka Yamamoto, Yuri Manabe, Mitsumasa Murao, Makiko Murota, Nobuhiro Kanaji, Yoshihiro Nishiyama

**Affiliations:** 1 Department of Radiology, Faculty of Medicine, Kagawa University, 1750-1 Ikenobe, Miki-cho, Kita-gun, Kagawa, 761-0793 Japan; 2 Division of Hematology, Rheumatology, and Respiratory Medicine, Department of Internal Medicine, Faculty of Medicine, Kagawa University, Miki-cho, Kagawa, Japan

**Keywords:** Molecular medicine, Oncology

## Abstract

Identifying the epidermal growth factor receptor (EGFR) mutation status is important for the optimal treatment of patients with EGFR mutations. We investigated the relationship between ^18^F-fluorodeoxyglucose (FDG) positron emission tomography (PET) texture indices and EGFR mutation status in patients with newly diagnosed lung adenocarcinoma. We retrospectively analyzed data of patients with newly diagnosed lung adenocarcinoma who underwent pretreatment FDG PET/computed tomography and EGFR mutation testing between August 2014 and November 2020. Patients were divided into mutated EGFR and wild-type EGFR groups. The maximum standardized uptake value (SUVmax) and 31 texture indices for the primary tumor were calculated from PET images and compared between the two groups. Of the 66 patients included, 22 had mutated EGFR and 44 had wild-type EGFR. The SUVmax did not significantly differ between the two groups. Among the 31 evaluated texture indices, the following five showed a statistically significant difference between the groups: correlation (*P* = 0.003), gray-level nonuniformity for run (*P* = 0.042), run length nonuniformity (*P* = 0.02), coarseness (*P* = 0.006), and gray-level nonuniformity for zone (*P* = 0.04). Based on the preliminary results of this study in a small patient population, FDG PET texture indices may be potential imaging biomarkers for the EGFR mutation status in patients with newly diagnosed lung adenocarcinoma.

## Introduction

Non-small cell lung cancer (NSCLC) is the most common type of lung cancer and remains the leading cause of cancer-related death worldwide^1^. The most common NSCLC subtype is adenocarcinoma, in which epidermal growth factor receptor (EGFR) mutations are one of the most widely recognized genomic alterations^1^. EGFR-targeted tyrosine kinase inhibitors have proven to be one of the most effective therapeutic options for patients with NSCLC carrying EGFR mutations^2^. Therefore, identifying the EGFR mutation status is important for the optimal treatment of patients with NSCLC. Although biopsy is the gold standard for gene mutation diagnosis, it is difficult to obtain satisfactory specimens due to various factors. Consequently, it is important to develop simple and noninvasive methods to identify the EGFR mutation status.

Studies in the field of imaging genomics have demonstrated the potential of imaging biomarkers for determining tumor genotype^3^. Positron emission tomography (PET) with ^18^F-fluorodeoxyglucose (FDG) is a very useful molecular imaging technique for staging, restaging, and prediction of tumor response in NSCLC^4^. Although several studies have evaluated the association between the EGFR mutation status and FDG PET parameters, such as the maximum standardized uptake value (SUVmax), which is the most commonly used in clinical practice, the results are still controversial, and the relationship is not yet firmly established^5–11^. In our recent study on NSCLC, the SUVmax also failed to identify the EGFR mutation status^10^.

In recent years, beyond simple measurements of tumor intensity of radioactivity, such as SUVmax, there is growing recognition of measures of tumor heterogeneity. To the best of our knowledge, to date, few published studies have focused on the tumor heterogeneity from PET images for identifying the EGFR mutation status^11–15^. Therefore, we evaluated the relationship between FDG PET texture indices and the EGFR mutation status in patients with newly diagnosed lung adenocarcinoma.

## Results

Complete data were available for 91 patients. Of these, 22 were excluded due to insufficient FDG uptake in the primary tumor for texture analysis, and three were excluded due to the presence of anaplastic lymphoma kinase rearrangement. Finally, 66 patients (41 men, 25 women; mean age, 73 years; age range, 42–92 years) were included in the study.

Tissue specimens for EGFR testing were obtained by surgical resection in 21 and by biopsy in 45 patients. There were 22 patients in the mutated EGFR group and 44 in the wild-type EGFR group. The patients’ clinical characteristics according to the EGFR mutation status are summarized in Table 1.

**Table 1:** Patients’ characteristics.

Characteristic	Mutated EGFR (n = 22)	Wild-type EGFR (n = 44)
Age (years)		
Mean	73	73
Range	59–86	42–92
Sex		
Male	11	30
Female	11	14
Clinical stage		
I	5	3
II	1	3
III	1	10
IV	15	28
Smoking history		
Smoking	6	32
Non-smoking	16	12

*EGFR* epidermal growth factor receptor.

Table 2 shows the relationship between FDG PET parameters and the EGFR mutation status. Although the SUVmax in the mutated EGFR group was lower than that in the wild-type EGFR group, this difference was not significant (*P* = 0.23). Among the 31 evaluated texture indices, five showed a statistically significant difference between the groups: correlation (*P* = 0.003), gray-level nonuniformity for run (*P* = 0.042), run length nonuniformity (*P* = 0.02), coarseness (*P* = 0.006), and gray-level nonuniformity for zone (*P* = 0.04). The area under the curve (AUC) values obtained via receiver operating curve (ROC) analysis of FDG PET parameters to discriminate between the mutated EGFR group and wild-type EGFR group are shown in Table 3.

**Table 2:** Relationship between FDG PET parameters and EGFR mutation status in patients with newly diagnosed lung adenocarcinoma.

FDG PET parameter	Mutated EGFR (n = 22)	Wild-type EGFR (n = 44)	*P* value
SUVmax	11.65 ± 5.09	13.31 ± 6.21	0.23
Homogeneity	0.30 ± 0.10	0.34 ± 0.13	0.32
Energy	0.013 ± 0.012	0.019 ± 0.030	0.38
Contrast	47.00 ± 30.13	48.76 ± 42.47	0.86
**Correlation**	**0.30 ± 0.10**	**0.42 ± 0.15**	**0.003**
Entropy	2.10 ± 0.35	2.15 ± 0.41	0.58
Dissimilarity	5.04 ± 2.06	4.85 ± 2.48	0.76
SRE	0.95 ± 0.03	0.93 ± 0.06	0.15
LRE	1.21 ± 0.16	1.41 ± 0.58	0.12
LGRE	0.0052 ± 0.0078	0.0070 ± 0.0156	0.62
HGRE	678.5 ± 495.5	857.2 ± 711.3	0.29
SRLGE	0.0048 ± 0.0069	0.0057 ± 0.0117	0.73
SRHGE	655.1 ± 482.1	811.5 ± 657.9	0.32
LRLGE	0.0073 ± 0.0125	0.0172 ± 0.0534	0.39
LRHGE	780.7 ± 553.1	1189.6 ± 1,493.1	0.22
**GLNUr**	**16.58 ± 10.11**	**46.82 ± 67.64**	**0.042**
**RLNU**	**222.0 ± 201.1**	**454.5 ± 426.4**	**0.02**
RP	0.94 ± 0.04	0.91 ± 0.08	0.11
**Coarseness**	**0.025 ± 0.010**	**0.016 ± 0.011**	**0.006**
Contrast	0.47 ± 0.27	0.43 ± 0.37	0.72
Busyness	0.26 ± 0.24	0.77 ± 2.39	0.32
SZE	0.64 ± 0.17	0.61 ± 0.18	0.48
LZE	38.16 ± 89.71	1498 ± 7707	0.38
LGZE	0.0053 ± 0.0083	0.0066 ± 0.015	0.71
HGZE	652.6 ± 476.3	834.6 ± 659.7	0.25
SZLGE	0.0025 ± 0.0029	0.0020 ± 0.0025	0.44
SZHGE	455.8 ± 352.6	588.2 ± 534.6	0.30
LZLGE	0.800 ± 2.34	104.8 ± 627.0	0.44
LZHGE	5746 ± 4163	76,291 ± 188,251	0.09
**GLNUz**	**7.20 ± 4.84**	**12.13 ± 10.13**	**0.04**
ZLNU	60.03 ± 67.04	85.45 ± 71.56	0.17
ZP	0.49 ± 0.20	0.42 ± 0.21	0.24

Data are presented as mean ± standard deviation. Significant differences are indicated in bold font. *FDG PET* ^18^F-fluorodeoxyglucose, *PET* positron emission tomography, *EGFR* epidermal growth factor receptor, *SUVmax* maximum standardized uptake value, *SRE* short-run emphasis, *LRE* long-run emphasis, *LGRE* low gray-level run emphasis, *HGRE* high gray-level run emphasis, *SRLGE* short-run low gray-level emphasis, *SRHGE* short-run high gray-level emphasis, *LRLGE* long-run low gray-level emphasis, *LZHGE* long-zone high gray-level emphasis, *GLNUr* gray-level non-uniformity for run, *RLNU* run length nonuniformity, *RP* run percentage, *SZE* short-zone emphasis, *LZE* long-zone emphasis, *LGZE* low gray-level zone emphasis, *HGZE* high gray-level zone emphasis, *SZLGE* short-zone low gray-level emphasis, *SZHGE* short-zone high gray-level emphasis, *LZLGE* long-zone low gray-level emphasis, *LZHGE* long-zone high gray-level emphasis, *GLNUz* gray-level non-uniformity for zone, *ZLNU* zone length nonuniformity, *ZP* zone percentage.

**Table 3:** Predictive performance of FDG PET parameters for determining EGFR mutation status.

FDG PET parameter	AUC (95% CI)	Sensitivity	Specificity	Accuracy
Correlation	0.753 (0.638–0.868)	0.659	0.773	0.697
GLNUr	0.682 (0.552–0.812)	0.545	0.773	0.621
RLNU	0.713 (0.583–0.843)	0.705	0.727	0.712
Coarseness	0.733 (0.608–0.859)	0.727	0.659	0.682
GLNUz	0.662 (0.530–0.795)	0.727	0.591	0.682

*EGFR* epidermal growth factor receptor, *FDG* ^18^F-fluorodeoxyglucose, *PET* positron emission tomography, *AUC* area under the curve, *CI* confidence interval, *GLNUr* gray-level non-uniformity for run, *RLNU* run length nonuniformity, *GLNUz* gray-level non-uniformity for zone.

**Figure 1:**
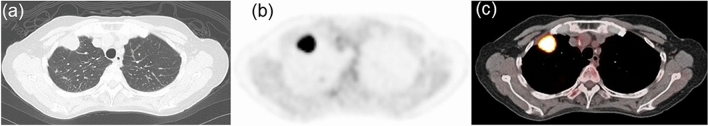
Representative images of a 69-year-old woman with mutated EGFR lung adenocarcinoma. (**a**) CT image showing a mass in the right upper lobe. (**b**) FDG PET and (**c**) PET/CT fusion images showing intense uptake in the tumor (SUVmax = 16.80, correlation = 0.10, GLNUr = 6.86, RLNU = 136.6, coarseness = 0.033, LZHGE = 5223, and GLNUz = 4.25). *CT* computed tomography; PET, positron emission tomography, *FDG* ^18^F-fluorodeoxyglucose, *EGFR* epidermal growth factor receptor, *SUVmax* maximum standardized uptake value, *GLNUr* gray-level nonuniformity for run, *RLNU* run length nonuniformity, *LZHGE* long-zone high gray-level emphasis, *GLNUz* gray-level nonuniformity for zone.

**Figure 2:**
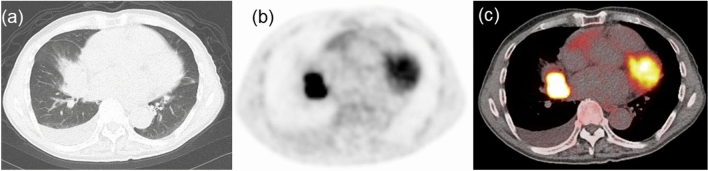
Representative images of a 77-year-old man with wild-type EGFR lung adenocarcinoma. (**a**) CT image showing a mass in the right middle lobe. (**b**) FDG PET and (**c**) PET/CT fusion images showing intense uptake in the tumor (SUVmax = 17.72, correlation = 0.45, GLNUr = 13.14, RLNU = 312.8, coarseness = 0.017, LZHGE = 6505, and GLNUz = 8.31). *CT* computed tomography, *PET* positron emission tomography, *FDG* ^18^F-fluorodeoxyglucose, *EGFR* epidermal growth factor receptor, *SUVmax* maximum standardized uptake value, *GLNUr* gray-level nonuniformity for run, *RLNU* run length nonuniformity, *LZHGE* long-zone high gray-level emphasis, *GLNUz* gray-level nonuniformity for zone.

Representative PET/computed tomography (CT) images from the mutated EGFR and wild-type EGFR groups are shown in Figs. 1 and 2, respectively.

## Discussion

In the present study, we found that five FDG PET texture indices, but not SUVmax, were related with the EGFR mutation status in patients with newly diagnosed lung adenocarcinoma.

Tyrosine kinase inhibitors targeting EGFR mutations have proven to be one of the most effective therapeutic options currently available, and EGFR mutations predict a favorable prognosis in patients treated with them^2^. However, previous studies using the FDG SUVmax to predict the EGFR mutation status have reported inconsistent findings. Mak et al.^8^ and Caicedo et al.^9^ found no significant association between the EGFR mutation status and SUVmax, which is consistent with the findings of the present study. Zhang et al.^11^ and Cho et al.^5^ indicated that tumors with a lower SUVmax tended to have EGFR mutations. All patients enrolled in the four aforementioned studies had NSCLC. Conversely, Ko et al.^6^ and Huang et al.^7^ reported that a higher SUVmax was a predictor of EGFR mutations. However, these two studies included only patients with lung adenocarcinoma, which has been reported to have a high EGFR mutation rate^16^. This discrepancy in the findings may be due to differences in the sample size, patient selection criteria, and methodology among these studies. Furthermore, the inconsistent findings may be attributed to the intratumor heterogeneity in NSCLC. SUVmax, which reflects the highest FDG uptake within the tumor, is the value of a single voxel within the region. Texture indices may be able to reflect more metabolic information on tumor behaviors than SUVmax, such as intratumor metabolic heterogeneity and genetic mutation status.

Intratumor metabolic heterogeneity is a key sign of tumor development and reflects the molecular biology or genetic alterations during tumor evolution^17^. In the present study, 5 out of 31 texture indices were significantly different between the mutated EGFR and wild-type EGFR groups in patients with newly diagnosed lung adenocarcinoma. Yip et al. investigated the relationship between the EGFR mutation status and 19 FDG PET radiomic features in 348 patients with NSCLC, and they showed that eight radiomic features were related to the EGFR mutation status^13^. Zhang et al. examined the intratumor heterogeneity among various subtypes of NSCLC through multi-region tissue sequencing and concluded that EGFR-mutant lung adenocarcinoma has the highest intratumor heterogeneity compared with that of other NSCLC subtypes^14^. Zhang et al. investigated the utility of FDG PET and CT radiomic features for discriminating the EGFR mutation status in NSCLC^11^. Although CT and PET alone radiomic models had a better predictive performance than SUVmax, the combined PET/CT radiomic model further improved the predictive performance for the EGFR mutation status^11^. Another study also showed that FDG PET/CT-based radiomic features, comprising two PET and four CT features, had good performance in predicting the EGFR mutation in NSCLC^12^. In their study, the diagnostic accuracies of PET radiomics, CT radiomics, and PET/CT radiomics for EGFR mutation status were 0.712, 0.753, and 0.771, respectively^12^. In our study, the accuracy of FDG PET texture indices ranged from 0.621 to 0.712. Yamazaki et al. evaluated 14 intratumoral and 18 peritumoral CT radiomics for the prediction of EGFR mutation in lung cancer^18^. The AUCs of intratumoral CT radiomics and combined intratumoral and peritumoral CT radiomics were 0.730 and 0.774, respectively^18^. In our study, the AUCs of FDG PET texture indices ranged from 0.662 to 0.753. Their results were not comparable to our findings, but it is difficult to compare them due to the different methodologies used. Shi et al. calculated the coefficient of variation as a heterogeneity index in NSCLC and found that a high coefficient of variation was significantly related to EGFR mutations^15^. Although these studies have investigated intratumor heterogeneity, the calculation methods vary across studies. At present, there are limited reports available on the association between intratumor glucose metabolic heterogeneity and EGFR mutation status. Orlhac et al. observed that healthy tissue showed higher homogeneity, lower entropy, higher low gray-level zone emphasis, and lower high gray-level zone emphasis than tumor tissue on FDG PET^19^. Chan et al. reported that the parameters of FDG PET heterogeneity such as coarseness, contrast, and busyness were associated with overall survival in patients with pharyngeal carcinoma^20^. Many texture indices have been reported as potentially useful; however, there is no clear indication as to which one should be used. To understand these texture indices, it is essential to carefully investigate their relationship with actual tumor characteristics.

There are several limitations to this study. First, it was retrospective in design with a small sample size. Second, the EGFR mutation status was investigated only in one lung cancer type (adenocarcinoma); thus, further studies in other lung cancer types are warranted. Third, although patients with co-mutations were excluded from this study, not all oncogenes could be evaluated. Fourth, we only analyzed FDG PET parameters. Although the optimal threshold of tumor volume for texture analysis varies across studies, previous studies have suggested that combining promising parameters, such as PET and CT, may be helpful for identifying the EGFR mutation status^11,12^. Therefore, further studies with a larger number of patients are needed to explore the role of FDG PET reflecting intratumor metabolic heterogeneity in identifying the EGFR mutation status, which can be very important for the selection of targeted therapies in clinical practice.

In conclusion, our preliminary findings in a small patient population indicated that FDG PET texture indices may be potential imaging biomarkers for the EGFR mutation status in patients with newly diagnosed lung adenocarcinoma, although the mechanism and biological significance remain unclear. Further prospective studies with bigger sample sizes will help to clarify the utility of FDG PET as an alternative indicator of EGFR mutation status when tissue samples are not available.

## Methods

### Study design and population

We reviewed the records of patients with newly diagnosed lung adenocarcinoma who underwent pretreatment FDG PET/CT and EGFR mutation testing in tumor tissue specimens from August 2014 to November 2020. Patients with incomplete data, insufficient image quality, and co-mutations were excluded.

The study was conducted in accordance with ethical standards of the Helsinki declaration in 1964 and its later amendments. This study was approved by the Ethics Committee of the Faculty of Medicine, Kagawa University (approval numbers: 2022-126), and a waiver for the requirement for written informed consent was granted because of the retrospective observational study design.

### FDG PET/CT imaging and analysis

FDG was produced by an automated synthesis system equipped with HM-18 cyclotron (QUPID; Sumitomo Heavy Industries Ltd, Tokyo, Japan). PET/CT was performed using a Biograph mCT 64-slice PET/CT scanner (Siemens Medical Solutions USA Inc., Knoxville, TN, USA). The patients fasted for at least 5 h before FDG injection. A normal glucose level was confirmed before intravenous injection of FDG (5.5 MBq/kg). Emission data were obtained after 90 min of rest, ranging from the mid-cranium to the proximal thighs (2 min per bed position). Non-contrast low-dose CT of the same area was performed for attenuation correction and image fusion. PET data were reconstructed using a Gaussian filter with an ordered subset expectation maximization algorithm, incorporating a correction with point-spread function and time-of-flight model (two iterations, 21 subsets).

A board-certified nuclear medicine radiologist performed the PET/CT image analysis. A volume of interest of the primary tumor was selected using a threshold of 40% SUVmax. The SUVmax and 31 texture indices for the primary tumor were calculated using the LIFEx package^21^. Texture indices were extracted from four different matrices computed for each volume of interest: gray-level co-occurrence matrix, gray-level run length matrix, neighborhood gray-level difference matrix, and gray-level zone length matrix (Table 4)^22^.

**Table 4:** Texture indices.

Matrix	Index
Gray-level co-occurrence matrix (GLCM)	Homogeneity
	Energy
	Contrast
	Correlation
	Entropy
	Dissimilarity
Gray-level run length matrix (GLRLM)	Short-run emphasis (SRE)
	Long-run emphasis (LRE)
	Low gray-level run emphasis (LGRE)
	High gray-level run emphasis (HGRE)
	Short-run low gray-level emphasis (SRLGE)
	Short-run high gray-level emphasis (SRHGE)
	Long-run low gray-level emphasis (LRLGE)
	Long-run high gray-level emphasis (LRHGE)
	Gray-level nonuniformity for run (GLNUr)
	Run length nonuniformity (RLNU)
	Run percentage (RP)
Neighborhood gray-level difference matrix (NGLDM)	Coarseness
	Contrast
	Busyness
Gray-level zone length matrix (GLZLM)	Short-zone emphasis (SZE)
	Long-zone emphasis (LZE)
	Low gray-level zone emphasis (LGZE)
	High gray-level zone emphasis (HGZE)
	Short-zone low gray-level emphasis (SZLGE)
	Short-zone high gray-level emphasis (SZHGE)
	Long-zone low gray-level emphasis (LZLGE)
	Long-zone high gray-level emphasis (LZHGE)
	Gray-level nonuniformity for zone (GLNUz)
	Zone length nonuniformity (ZLNU)
	Zone percentage (ZP)

### EGFR mutation testing

Tissue specimens of the primary tumors were obtained by surgical resection or biopsy. EGFR mutation testing was performed using the Cobas^®^ EGFR Mutation Test v2 (Cobas; Roche Diagnostics, Basel, Switzerland). Based on the EGFR mutation status, patients were divided into mutated EGFR and wild-type EGFR groups.

### Statistical analysis

Differences in PET parameters between the two groups were analyzed using a logistic regression method. ROC analyses were performed and AUC values were determined to evaluate the diagnostic ability of the FDG PET parameters for discriminating between the mutated EGFR and wild-type EGFR groups. IBM SPSS Statistics version 26 (IBM Corp., Armonk, NY, USA) was used for the analysis. Differences were considered statistically significant at a *P* value of less than 0.05.

## Data Availability

The datasets analyzed in during the current study are available from the corresponding author on reasonable request.
